# A Case of Hyperimmunoglobulinemia D Syndrome Successfully Treated with Canakinumab

**DOI:** 10.1155/2013/795027

**Published:** 2013-04-16

**Authors:** Elena Tsitsami, Charis Papadopoulou, Matthaios Speletas

**Affiliations:** ^1^Pediatric Rheumatology Unit, 1st Department of Pediatrics, Children's Hospital “Aghia Sophia”, University of Athens, 115 27 Athens, Greece; ^2^Department of Immunology & Histocompatibility, School of Medicine, University of Thessaly, 411 10 Biopolis, Larissa, Greece

## Abstract

Hyperimmunoglobulinemia D syndrome is a rare autosomal recessive autoinflammatory disorder caused by mutations in the mevalonate kinase gene (*MVK*). In a proportion of patients, however, no *MVK* mutations are detected. Although various standard anti-inflammatory drugs have been tried, until now there is no consensus about how HIDS should be treated. We present a case of HIDS in an 8-year-old girl whose clinical picture had started before the end of the first year of life. The patient had consistently elevated IgD levels but no mutations were found after a full-length analysis of the *MVK* gene. The method of *MVK* mutational analysis is presented in details. Treatment with canakinumab in a final single dose of 4 mg/kg every 4 weeks resulted in the disappearance of febrile attacks and a considerable improvement of patients' quality of life during a 12-month follow-up period. The drug has been well tolerated, and no side effects were observed.

## 1. Introduction

Hyperimmunoglobulinemia D and periodic fever syndrome (HIDS) is a rare autosomal recessive autoinflammatory disorder, characterized by recurrent febrile attacks associated with lymphadenopathy, abdominal pain, diarrhea, arthralgia, and skin rash [1, 2]. HIDS is caused by mutations in the mevalonate kinase gene (*MVK*) resulting in deficient activity of the mevalonate kinase enzyme [3, 4]. Until the discovery of the *MVK* gene as underlying HIDS, the presence of a high level of serum IgD was necessary to establish the diagnosis of HIDS, but since, several cases of HIDS with *MVK* mutations and normal IgD serum levels have been described [3, 5]. On the other hand, Ammouri et al. [6] described 14 patients who had a recurrent fever syndrome with high level of IgD without *MVK* mutations and proposed to use the term of mevalonate kinase deficiency (MKD) for patients who have a biochemical deficiency of mevalonate kinase and *MVK* mutations and the term HIDS for the group of patients who have a recurrent fever syndrome and an elevated IgD concentration. A wide variety of immunomodulatory drugs was tried to treat and prevent attacks in these patients, including colchicine, steroids, and nonsteroidal anti-inflammatory drugs. Anakinra, a IL-1 receptor antagonist, has also been tested in a small number of patients [7–9].

We are reporting a case of HIDS in an 8-year-old girl treated with canakinumab, a newer IL-1 receptor antagonist, with an excellent response.

## 2. Case Presentation

An 8-year-old girl of central Greek ancestry was admitted to the hospital because of periodic fever spikes, which occurred every 3-4 weeks and lasted 3–5 days. Since the first months of life she had experienced characteristic febrile attacks. She had a surgery history of tonsillectomy and adenoidectomy at the age of 2 and 6, respectively. During fever flare ups, she usually developed chills, malaise, and abdominal pain with diarrhoea and vomiting. Between attacks the patient was free of symptoms. From her family history, recurrent febrile episodes during childhood were reported both to her mother and maternal grandmother. 

On admission, her blood pressure and temperature were normal. Physical examination showed normal findings, except from small aphthous ulcers in her mouth. No symptoms suggestive of respiratory, abdominal, or urinary infection were apparent. Chest radiography and abdominal ultrasonography scan revealed no abnormalities. Laboratory investigations revealed marked increase of erythrocyte sedimentation rate and C-reactive protein, a total leukocyte count of 5.710/mm^3^, serum amyloid 29.7 mg/L, serum IgD 80 mg/dL (normal range 0.3–11), and creatinine 0.37 mg/dL. Immunological tests, including antinuclear antibodies, rheumatoid factors, complement factors, serum immunoglobulins as well as serology for CMV, parvovirus, B19, EBV, adenovirus, coxsackie virus, and antistreptolysin O titre resulted negative or within normal limits. Common causes of infections were ruled out. The genetic analysis for familial Mediterranean fever (FMF) and cryopyrin-associated periodic syndromes (CAPS) was negative. A full-length analysis of the *MVK* gene was performed according to the method described below but no mutation was found.

Serum IgD was repeated several times and was always elevated. On this basis, the diagnosis of HIDS was considered and the patient started the therapy with canakinumab, an interleukin-1 receptor antagonist. Initially, a single dose (2 mg/kg) every 4 weeks was administered and resulted in prompt remission of the fever the other symptoms (abdominal pain, diarrhoea, and vomiting). Before, however, the third monthly dose of canakinumab the patient presented a new febrile episode with severe abdominal symptoms and, therefore, duplication of the dose was decided. Thereafter and during a followup of 12 months, the patient continues receiving canakinumab 4 mg/kg per month and remains free of febrile attacks. A considerable improvement of her quality of life was observed but the levels of acute phase reactants, although significantly diminished, remain constantly higher than normal values. The drug has been well tolerated and no side effects were observed. 

### 2.1. Mutational Analysis of *MVK* Gene

Genomic DNA was extracted from peripheral blood using the QIAamp DNA Blood Mini Kit (Qiagen, UK), according to manufacturer's instructions. Afterwards, an amplification of all 10 (ten) exons, including exon-intron boundaries, of *MVK* gene was performed. The position and the sequences of the utilized primers, designed with the aid of the Oligo-6 software (NBI, Plymouth, MN, USA), as well as the conditions of PCR reactions, are shown in Table 1. For each PCR reaction, a total of 100–200 ng of genomic DNA was amplified in a 30 *μ*L reaction using 62.5 *μ*M of each deoxynucleotide triphosphate, 20 pmoles of each primer and 0.8 U Taq Polymerase (Bioline, UK) in a buffer supplied by the manufacturer. The concentration of MgCl_2_ used in every PCR reaction is also provided in Table 1. All PCR procedures were carried out in the PCR-engine apparatus PTC-200, MJ-Research (Watertown-Massachusetts, USA), and the PCR products were analyzed in 2% TBE agarose gels, stained with ethidium bromide and visualized under UV light. Afterwards, PCR products were purified by the QIAquick gel extraction kit (Qiagen) and directly sequenced using an ABI Prism 310 Genetic Analyzer (Applied Biosystems, Foster City, CA, USA) and a Big Dye Terminator DNA sequencing kit (Applied Biosystems).

## 3. Discussion

HIDS is characterized by recurrent, self-limiting attacks of fever occurring since early childhood. Febrile episodes usually last 3–7 days and are usually associated with headache, arthralgias, lymphadenopathy, abdominal pain, diarrhoea, vomiting, and skin lesions. The causative mutations in HIDS are located in the gene encoding *MVK*, an enzyme in the isoprenoid metabolism, the end-products of which include cholesterol, protein isoprenylation, dolichol, and ubiquinone [10]. 

In our patient, the recurrent and periodic nature of her illness over a period of 7 years was suggestive of a periodic fever syndrome. A number of periodic fever syndromes were excluded based on a combination of laboratory findings, genetic testing, and clinical features. Although no test for* TNFRSF1A* mutation was performed, the patient's clinical features and the lack of family history for an autosomal dominant disorder made TRAPS defect to seem unlikely. Serum IgD values were >11 mg/dL on more than two occasions one month apart. Although this does not completely confirm the diagnosis of HIDS, again the periodicity and patterns of fever and the clinical symptoms in our patient were very suggestive for this syndrome. 

The exact pathogenesis of HIDS is unclear. However, several of the isoprenoid end-products have been linked with apoptosis [11]. Evidence exists for a pathologic role of IL-1*β* activation through caspase-1 [12] and for successful treatment with anakinra in acute crises [7–9]. Treatment of monocytes with simvastatin, an inhibitor of isoprenoid biosynthesis mimicking MKD, resulted in increased activation of caspase-1-mediated IL-1*β* activation [13]. Canakinumab, another IL-1 receptor antagonist, has been administered to our patient on the basis of its excellent results in patients with other IL-1-mediated syndromes [14]. The pharmacokinetics of this drug is in favour of a good compliance of patients to the long-term treatment although sustained IL-1 suppression may have potentially serious long-term effects, which are currently unknown. 

Autoinflammatory syndromes always pose diagnostic and therapeutic challenges to treating clinicians. The clinical description of the diversity of periodic fever syndromes is helpful in the assessment and management of these patients. Our case confirms the previous reports indicating that IL-1 targeting drugs brings substantial benefit to HIDS patients [7, 8]. To the best of our knowledge this is the first report of HIDS treatment with canakinumab indicating that controlled trials are worthy to be undertaken to further assess its clinical benefit and safety in these patients.

## Figures and Tables

**Table 1:** Primers and PCR conditions used for the detection of *MVK* mutations.

Exon(s)	Primer	Sequence	PCR conditions	PCR product
1	forward reverse	5′-GAGTGGAAGAGCTGGCATTTGAA-3′ 5′-GCCTCAGGGTGTCCTTTTACCTTCT-3′	94°C for 2 min, followed by 32 cycles (94°C for 30 s, 56°C for 30 s, 72°C for 30 s) and a final elongation at 72°C for 5 min. MgCl_2_: 1.5 mM	381 bp
2	forward reverse	5′-TGGCCTCTGTGCTTATGTTTG-3′ 5′-AAGCTAATTTTATGAGGTCAGGGTA-3′	94°C for 2 min, followed by 30 cycles (94°C for 30 s, 58°C for 30 s, 72°C for 30 s) and a final elongation at 72°C for 5 min. MgCl_2_: 1.5 mM	386 bp
3	forward reverse	5′-AAAGTCCCTCTCACCCACTTGTGTT-3′ 5′-CCAGCAGCAATGACAGAAACTCTTC-3′	94°C for 2 min, followed by 30 cycles (94°C for 30 s, 55°C for 30 s, 72°C for 45 s) and a final elongation at 72°C for 5 min. MgCl_2_: 2.5 mM	271 bp
4	forward reverse	5′-GAATTTGAAGACGCACATTATTACA- 3′ 5′-GGCCACTATGACACCTCTCAC-3′	94°C for 2 min, followed by 32 cycles (94°C for 30 s, 55°C for 30 s, 72°C for 70 s) and a final elongation at 72°C for 5 min. MgCl_2_: 1.5 mM	1010 bp
5 & 6	forward reverse	5′-AGCTGGAGAGGTTCAGAGTGGACTT-3′ 5′-GGGAGAAGGAGAGAGCAGGTCT-3′	94°C for 2 min, followed by 32 cycles (94°C for 30 s, 58°C for 30 s, 72°C for 70 s) and a final elongation at 72°C for 5 min. MgCl_2_: 2.0 mM	1116 bp
7 & 8	forward reverse	5′-TGCCTCCCTTCTTCCTCCGTATC-3′ 5′-CCTCCCTTGCACTCTCCCAATTACT-3′	94°C for 2 min, followed by 30 cycles (94°C for 30 s, 57°C for 30 s, 72°C for 70 s) and a final elongation at 72°C for 5 min. MgCl_2_: 1.5 mM, 10% DMSO	932 bp
9	forward reverse	5′-GTCTCCAGCCAACAACTGTCAGATG-3′ 5′-TTCCAGGTCTCTTTTTCCTTTCAA-3′	94°C for 2 min, followed by 30 cycles (94°C for 30 s, 57°C for 30 s, 72°C for 30 s) and a final elongation at 72°C for 5 min. MgCl_2_: 2.5 mM	352 bp
10	forward reverse	5′-TTGCCTTGAATATGATGAGCTTC-3′ 5′-GCCAGCACAGAGTCGAACTG-3′	94°C for 2 min, followed by 30 cycles (94°C for 30 s, 57°C for 30 s, 72°C for 30 s) and a final elongation at 72°C for 5 min. MgCl_2_: 2.5 mM	306 bp
